# Correction: Integrated bioinformatics and statistical approach to identify the common molecular mechanisms of obesity that are linked to the development of two psychiatric disorders: Schizophrenia and major depressive disorder

**DOI:** 10.1371/journal.pone.0304339

**Published:** 2024-05-21

**Authors:** The PLOS One Staff

## Notice of Republication

This article was republished on May 10^th^, 2024, to correct errors in the title. Please download this article again to view the correct version. The publisher apologizes for the error. The originally published, uncorrected article and the republished, corrected articles are provided here for reference.

## Supporting information

S1 File Originally published, uncorrected article. (PDF)

S2 File Republished, corrected article. (PDF)
