# Supplementary material for: Integrated bioinformatics and statistical approach to identify the common molecular mechanisms of obesity that are linked to the development of two psychiatric disorders: Schizophrenia and major depressive disorder
Source: PLoS One. 2023 Jul 26;18(7):e0276820. doi: 10.1371/journal.pone.0276820 (PMC10370737; doi:10.1371/journal.pone.0276820)
Supplement: Attachment — Submitted filename: Review_Report_DrHMV.doc [file pone.0276820.s001.doc]

The manuscript entitled " Integrated Bioinformatics Approaches to Identify the Pathobiological Associations of Schizophrenia and Major Depressive Disorder with Obesity" investigates the genetic interaction of Obesity with Schizophrenia and Major Depressive Disorder. Given that people with Obesity are more likely to have Schizophrenia and Major Depressive Disorder, such studies can help to determine therapeutic targets to minimize the likelihood of the occurrence of the target diseases. Some gained results are very intriguing and significant but still, some concerns remain:

01. There are some inconsistencies like typo errors in the writing of the manuscript. Please address them.

02. There are several psychiatric disorders but Why the authors chose these two particular psychiatric disorders is not clear. The authors should explain more details in the introduction sections.

03. Although the authors have provided the rationale behind considering datasets from iPSCs samples. More description is required on the datasets in the materials and Methods sections.

04. The authors should provide relevant recent references in the introduction and discussion sections.

05. Discussion section needs to improve and need to compare with the recent similar work.

06. Which disease among the two psychiatric disorders is mostly affected by obesity? Need to discuss in the discussion section.

06. The reason behind clustering co-expressed genes should be more clear.

07. How sensitive is the method to a low sample size such as schizophrenia and major depressive disorder?

08. Line 33-39 is not clear

09. What's the reason behind taking the 4 highlighted cluster areas only?

10. Why threshold-based cluster analysis rather than k-means is used, the author should explain more details.
