# Supplementary material for: Integrated bioinformatics and statistical approach to identify the common molecular mechanisms of obesity that are linked to the development of two psychiatric disorders: Schizophrenia and major depressive disorder
Source: PLoS One. 2023 Jul 26;18(7):e0276820. doi: 10.1371/journal.pone.0276820 (PMC10370737; doi:10.1371/journal.pone.0276820)
Supplement: Attachment — Submitted filename: Review comments.docx [file pone.0276820.s002.docx]

“Integrated Bioinformatics Approaches to Identify the Pathobiological Associations of Schizophrenia and Major Depressive Disorder with Obesity”. The author applied public datasets of transcriptome and GWAS/WGS to investigate the shared genes and pathways between obesity and SZ, as well as obesity and MDD. The idea of the study is interesting; however, the results are not strong enough to support their conclusion.

Major issues:

1. Obesity could not be considered as a major risk of SZ. The obesity existed in SZ patients is more likely due to the use of psychotropic medications such as second-generation antipsychotics. Hence, the transcriptome and genetic association study between obesity and SZ should be take a careful consideration.
2. The author involved iPSC from obesity subjects, and these iPSC were differentiated into hypothalamic neurons. Hypothalamic is a central coordinator of obesity. However, the iPSC used for SZ and MDD were finally differentiated into forebrain neurons. Actually, the authors compared the differently expressed genes between different neurons. Although they found some overlapped genes, especially those changed in the same direction, those genes could be functioned in the basic biological process of neurons. In fact, the GO enrichment and pathway results in this study also showed this problem---they are mostly enriched in synaptic associated processes. Hence, such comparison could not provide comprehensive evidence about the common biological mechanism shared between obesity and psychiatric disorders.
3. The GWAS and WGS data might provide more convincing association between obesity and psychiatric disorders, however, there are not many results and interpretation about these datasets. In addition, the analysis method of this part is not clear and missing important description. For example, when combing the large dataset for analysis, how the author handled with the confounding factors, such as ethnicity, age, sex, drug dosage, smoking and others?

Other issues:

Overall, the author needs to revise the language to improve readability.

Title: the title is confusing. It seems to study the samples of schizophrenia with obesity and samples of MDD with obesity.

Keywords: too much keywords. “comorbidities” is not relevant to this study, since subjects the author involved in are not patients comorbidity with any other disease.

Abstract:

1. “Obesity is a major risk factor for both schizophrenia and MDD.” The author need pay attention to the phrase of “a major risk factor”, especially for schizophrenia. There is no evidence indicate that obesity is a major risk factor for SZ. The author need double check this statement.
2. “… in patients with psychiatric disorders that are also relevant to the pathogenicity of obesity.” The author need correct this sentence. It makes readers to think these are patients comorbidity with obesity.
3. The last sentence “we also validated our findings by use of GWAS and WGS”. It is confused how the author validated and confirmed their findings? Since there is no any result showed the overlap between the DEGs found in transcriptomic analysis and those found in GWAS/WGS. “Confirmed the likely involvement” this statement is also ambiguous.

Introduction:

1^st^ p: Revise the paragraph to make it brief and clear for reader to understand obesity and the aim of study. Too much irrelevant information inside.

2^nd^ p: ref 17 and 18: no any information about the association between obesity and schizophrenia indicated. The author should correct the refs.

3^rd^-4^th^ p: The author needs involve more information about their association with obesity, not just explain the two diseases. Importantly, the author needs to explain why they choose two psychiatric disorders to analysis? What is their relevance?

5^th^ p, line 76: Please check the ref 17. It could not find any information about the Chinese ethnicity in this ref.

6^th^-7^th^ p: It is confused why the author talked much about endocrine systems with obesity and psychiatric disorders. How the endocrine systems relate to the study aim? In addition, line 91-97, this long sentence is very hard to read. Line 97-98: this sentence is also hard to read. How “these 70 candidate genes” comes from?

8^th^ p: line 107-109: it is very hard to follow the logic of this sentence.

9^th^ p: line 122: missing WGS data. In addition, the author should explain in the introduction what kind of data they were used in this study and why such iPSC data were selected for this study.

Method:

4. Page 7, line 166: the author need make some comments why they did not do the FDR correction for DEG analysis.
5. Page 7, line 168-177: Please make a more clear and detailed description about how this disease-gene network was conducted. It is hard to follow.
6. Page 7, section 2.2: Please make a more detailed description about the sample for GWAS and WGS. Such as how many datasets/samples are used, what are their biological source. What kind of mutation information was involved for analysis? In addition, line 197-199: it could not find any information about the source of WGS data. Importantly, when combing the large dataset for analysis, how the author handled with the confounding factors, such as ethnicity, age, sex, drug dosage, smoking and others?
7. Page 8, line 190: “so may influence the [46]”. Please complete the sentence.
8. Page 8, line 191: it was confused why the p-value criteria was different from the result. (1.0e-6 vs 1.0e-5)
9. Page 9, line 243-245: why the author also analyzed the gene data correlated between SZ and MDD? It seems not the study aim and there was also no any interpretation about the result.

Result:

1. The author need display each figure number beside the relevant result to make readers easy follow.
2. Page 10, line 27: “including that…” this sentence is hard to follow. How many genes changed in the same direction? What are their function and involved pathways? These genes could be the key genes that associated with obesity and psychiatric disorders.
3. Page 10, line 273: “obesity patient iPSCs”, the author should correct the statement. In the method, some obesity samples are from postmortem brains. In addition, line 271-275: the content of the two sentences is similar, keep one of them is better. Also, the last sentence is not clear, why the author still what to compared the genes between SZ and MDD? How this result is related to the study aim？
4. Page 10, line 278-280: the interpretation is hard to follow.
5. Figure 2B-C: It is not clear why the author split the results into two figures. The fold change and P-Value should display for every protein in the same figure. In addition, there are 52 overlapped genes in the Venn diagram. Figure B-D just involved 51 genes?
6. Page 11, Figure 3 description: “obesity with schizophrenia and major depressive disorder.” It makes readers to think the obesity patients are comorbidity with SZ and MDD.
7. Page 11, line 284-285: why the criteria changed, different from the method? (Page 8, line 192)
8. Page 11, line 288: what is the p-value for SZ and MDD analysis?
9. Page 11, section 3.2: how many significant genes were overlapped with the transcriptome data for obesity, SZ and MDD?
10. Page 11, section 3.3. line 304-306: confused about this result. Why the number is different from Figure 2A. How these 44 and 80 shared genes come from?
11. Page 11, section 3.3. Not clear about the meaning of this part. It seems another way to explain figure 2A. It could not see how the disease -gene network works. What is the enrichment p-value of the network?
12. Page 12, section 3.4: The author needs to make a detailed description about this result. What GO function and pathway they received? How many shared genes were used for analysis?
13. Page 13, line 346-347, line 352-353: could not find the relevant figures or supplement material.
14. Page 13, section 3.6: not clear how the correlation matrix generated. The author need make clear how many shared genes were used. These genes were shared among three disease or any two of them? What is the biological function of these correlated modules? In addition, why only positive correlated modules were selected?
15. The results from transcriptome and GWAS/WGS were displayed separately, what are their relevance. The author needs to make some comparison analysis.

Discussion:

1^st^ p: the author needs to make it brief and clear. Not clear why emphasis the endocrine system, how this related to the result and finding?

2^nd^ p: the author did not make a convincing explanation why the GWAS and WGS data did not perform as well as the transcriptome data. The author could discuss the impact of sample source for these datasets.

3^rd^ p: it is confused why the author discussed much about inflammation? There were no any results showed inflammation in this study. The author should make more discussion based on their findings (GO enrichment and pathways).

4^th^ p: the author needs to make more discussion about the limitation of the study.
